# Review of Recent Prevalence of Urogenital Schistosomiasis in Sub-Saharan Africa and Diagnostic Challenges in the Field Setting

**DOI:** 10.3390/life13081670

**Published:** 2023-07-31

**Authors:** Sung-Tae Hong

**Affiliations:** 1 Graduate School of International Development, Handong Global University, Pohang 37554, Republic of Korea; hst@snu.ac.kr; 2 Department of Tropical Medicine and Parasitology, Institute of Endemic Diseases Medical Research Center, Seoul National University College of Medicine, Seoul 03080, Republic of Korea

**Keywords:** *Schistosoma haematobium*, urogenital schistosomiasis, sub-Saharan Africa, diagnosis, urine microscopy, urine reagent strips, ultrasound scanning, echogenic snow sign

## Abstract

Human schistosomiasis is one of neglected tropical diseases that remain highly prevalent in sub-Saharan Africa (SSA). Human schistosomiasis is mainly caused by two species, *Schistosoma haematobium* and *S. mansoni*, leading to urogenital and intestinal schistosomiasis, respectively. The World Health Organization (WHO) recommends mass drug administration (MDA) with praziquantel as the primary method of global intervention. Currently, MDA with praziquantel covers over half of the target population in endemic SSA countries. However, an accurate diagnosis is crucial for monitoring and evaluating the effectiveness of MDA. The standard diagnosis of both urogenital and intestinal schistosomiasis relies on the microscopic identification of eggs. However, the diagnostic sensitivity of this approach is low, especially for light or ultra-light infections. This is because *Schistosoma* eggs are laid inside of the venous plexus of the urinary bladder or mesenteric vein, where the adult flukes live. Approximately half of the eggs circulate in the blood vessels or are packed in neighboring tissues, while the remaining half are expelled into the lumen of the urinary bladder or intestine intermittently when the blood vessels are ruptured. In the field setting, the accuracy of any diagnostic method is critical for proper management of the intervention. The present article reviews the recent prevalence of urogenital schistosomiasis in SSA and highlights the practical limitations of diagnostic methods such as urine microscopy, urine reagent strips, molecular diagnosis, and ultrasound scanning in the field setting. Despite continuous global efforts to eliminate schistosomiasis over the past 20 years, many areas still remain endemic in SSA. No single diagnostic approach achieves acceptable sensitivity and specificity in the field setting. Therefore, any field survey should employ a combination of these methods based on the purpose of the study to accurately monitor and evaluate urogenital schistosomiasis. Based on diagnostic values and a cost–benefit analysis, a urine reagent strip test can replace urine microscopy in the field setting. The WHO criteria by ultrasound diagnosis should be updated including the echogenic snow sign and contour distortion.

## 1. Introduction

Schistosomiasis is a human helminthiasis, acute or chronic, caused by the blood fluke *Schistosoma* spp. Human schistosomiasis is clinically categorized into two forms: urogenital and intestinal schistosomiasis. About 90% of the victims live in tropic or subtropic Africa. The World Health Organization (WHO) estimated that at least 236 million people required preventive chemotherapy (PC) in 51 endemic countries in 2019 [1].

There are six human-infecting species in the genus *Schistosoma*, but urogenital schistosomiasis (UGS) is caused only by *Schistosoma haematobium* and other species induce intestinal schistosomiasis (IS). UGS by *S. haematobium* and IS by *S. mansoni* are mixed endemic in most poor communities of Sub-Saharan Africa (SSA). Both diseases are transmitted to the human body by contacting contaminated water during daily life [1].

The WHO, the Schistosomiasis Control Initiative (SCI), global pharmaceutical companies, and several global and local non-governmental organizations (NGOs) are supporting the implementation of PC by mass drug administration (MDA) with praziquantel to eliminate both UGS and IS in endemic countries. The United Nations (UN) direction of Sustainable Developmental Goals (SDGs) proposed a roadmap achieving the elimination of schistosomiasis by 2030 [2].

The pharmaceutical company Merck KGaA has donated 1.5 billion tablets of praziquantel in partnership with the WHO to support the MDA for both UGS and IS [3]. The donation has enabled the coverage rates of PC for schistosomiasis to be over 60% for school-aged children (SAC) in most African countries, but the national coverage varied by country from 0.4% in South Sudan to 96.5% in Burundi in 2021 (Table 1) [4]. In the countries where armed conflicts last for several years like South Sudan, the data might not be correct because the control efforts by PC are limited.

The control programs by MDA mainly target SAC due to limited resources, but about half of the UGS and IS victims are in communities outside of schools who are least covered by PC. Many of them are children of pre-school age, and also there are many school-unregistered children of school age. Furthermore, some groups of adults with high risks are infected heavily, although adults are less infected than children [1]. The MDA-uncovered infected people cause the discharge of eggs and keep a re-infection force in the community. Those infected outsiders of the PC are the main hurdle to effectively eliminate schistosomiasis in most endemic SSA countries for achieving the SDGs by 2030. The WHO intends to achieve coverage of over 75% of SAC and encourages all partners to eliminate both UGS and IS under streamlining the UN SDGs. The coverage rates have increased continuously. The 75% overall coverage in SAC has been achieved but not in national coverage in many SSA countries (Table 1). There are many limitations in the control programs but uninterrupted MDA for all SAC in and out of schools may break the fetters in SSA.

The control or elimination program of schistosomiasis can begin by successful funding and the funding must be sustainable. When any country-owned self-funding program is developed, international programs may synergistically keep them sustainable. Of course, the programs should organize various items of the field setting for real implementation, such as work design, manpower training and supply, sampling and baseline survey, diagnosis, drug supply and distribution, chemotherapy, education, monitoring of adverse effects after chemotherapy, and evaluation of PC effects. The whole process must be complete for the successful implementation of any control program. All of the components are important, but the diagnostic measure in the field setting is especially critical for the program.

Field surveys of UGS or IS in a certain area give basic epidemiological information of the main target community and population before launching an elimination program. During or after implementation of the program, monitoring and evaluation (M&E) is essential for correct interpretation. The baseline survey and M&E require a quality diagnosis of UGS or IS. There are several kinds of diagnostic technologies for both UGS and IS, but the practical diagnostic measures are limited in the field setting because of their diagnostic values, compliance, and feasibility. The present article reviews the recent prevalence of UGS in SSA and challenges of current diagnosis in the field setting.

## 2. Recent Status of UGS in Sub-Saharan Africa

### 2.1. Overall Reports on Prevalence

Every year, hundreds of articles are published on schistosomiasis. Table 2 summarizes the recently reported prevalence of UGS in SSA that was indexed in PubMed [5,6,7,8,9,10,11,12,13,14,15,16,17,18,19,20,21,22,23,24,25,26,27,28,29,30,31,32,33,34,35,36,37,38,39,40,41,42,43,44,45,46,47,48,49,50,51,52,53,54,55,56,57,58,59,60,61,62,63,64,65,66,67,68,69,70,71,72,73,74,75,76,77,78,79,80,81,82] following a review of reports by Aula et al. [83] up to 2020. Most of these reports were cross-sectional studies describing the endemicity of UGS through parasitological surveys that detected *S. haematobium* eggs in urine samples using urine microscopy (UM). Although the reports were listed from most endemic countries in SSA, a higher number of reported surveys were found in Cameroon, Ethiopia, Ghana, Nigeria, Tanzania, and Zimbabwe (Table 2).

### 2.2. Mapping Reports

Some of the surveys reported national- or state-level mapping of UGS (Table 2). Mendes et al. [5] reported a 12.6% UGS prevalence through a mapping of 31,938 SAC in Angola in 2018–2019. One population-based survey of 19,250 SAC in Benin confirmed that UGS was predominant, with a 17.6% prevalence [8]. In Ethiopia, a mapping of 15,133 SAC in 2018–2019 reported a prevalence of 0.13% [21], while Camara et al. reported a prevalence of 4.2% among 10,434 SAC in 2014 in Gambia [27]. Similarly, mapping in Sudan reported a prevalence of 5.2% among 100,726 SAC in 2016–2017 [61], and a cross-sectional study in Tanzania reported a prevalence of 5.3% through a population-based mapping in 2006–2007 [72].

These mapping data have reported a relatively low prevalence, likely due to random statistical population-based sampling. Given the random sampling, the prevalence of UGS, reported as 4–5% in Gambia and Sudan by mappings, suggests medium to high endemicity in that area [27,61]. Even when a focal survey reports rather high prevalence within limited areas, neighboring communities show low endemicity in the real situation because UGS forms its ecology within a rather narrow range. In this context, the mapping reports from Angola of 12.6% and 17.6% in Benin among SAC suggest a high prevalence of UGS in the subjected zone [5,8]. These mapping reports can be extrapolated to estimate, rather accurately due to their statistical sampling, the number of infected individuals and the distribution of UGS in the surveyed states. Therefore, mapping studies or population-based surveys produce highly significant epidemiological data for better design of elimination programs. It is optimal to implement regular mapping of UGS and other infectious tropical diseases with continuous interventions in endemic countries.

### 2.3. Local Prevalence Reports

As shown in Table 2, most surveys screened SAC following WHO recommendations, which is a cost-effective approach and well reflects endemicity in the surveyed locality [5,6,7,8,9,10,11,12,13,14,15,16,17,18,19,20,21,22,23,24,25,26,27,28,29,30,31,32,33,34,35,36,37,38,39,40,41,42,43,44,45,46,47,48,49,50,51,52,53,54,55,56,57,58,59,60,61,62,63,64,65,66,67,68,69,70,71,72,73,74,75,76,77,78,79,80,81]. The egg-positive rates varied from 0 to 67.4%, but the reported prevalence rates mostly ranged from 10–20% between 2018–2022 (Table 2). An article reported a 0/228 egg-positive rate in Burkina Faso in 2021, which did not necessarily suggest no prevalence, but a low prevalence [9].

Recent prevalence reports of UGS in known endemic areas in SSA showed a definite reduction in most countries compared to those by Aula et al. [82] and Kalindra et al. [83]. These lowered rates are likely outcomes of extensive global PC under the UN direction of SDGs [2,3,4]. After the launch of the Millennium Development Goals (MDGs) by the UN in 2000 and ending in 2015, the impact on the elimination of tropical diseases was significant. Since 2016, the SDGs have taken over the task of disease control activities. The SDGs have identified neglected tropical diseases (NTDs) as major target diseases, alongside HIV/AIDS, tuberculosis, and malaria [2]. Currently, global activities supported by MDGs and successively by SDGs could have contributed to the reduction in the prevalence, deaths, and disabilities caused by major tropical diseases, including schistosomiasis [4].

Field surveys in endemic areas have continuously reported the persistent transmission of UGS in all of SSA (Table 2). Several articles have reported a high prevalence of over 50% positivity in recent local surveys, including 100% and 39.2% in Chad [14,15], 60.4% in Mozambique [40], 67.4% and 65.3% in Nigeria [45,54], and 59.7% and 66.7% in Senegal [55,56]. Most of these reports were from hotspots with high re-infection rates, but some were unrecognized hyperendemic foci. Additionally, all surveys conducted in Cameroon [10,11,12,13], Chad [14,15], Gabon [23,24,25], and Senegal [55,56] reported over 30% egg-positive rates. In endemic areas of these countries, UGS is still highly transmitted locally, although many previously known areas have become less endemic by repeated MDAs.

Taken together, UGS is still dominantly prevalent throughout SSA (Table 2) but the endemicity is slowly becoming less due to extensive PC with praziquantel. As summarized in Table 1, PC coverage is high in SAC, but the national coverage is still low in many countries. Furthermore, the COVID-19 pandemic has suspended or cancelled most of the intervention programs of NTDs, which may mean an actual increase in UGS prevalence compared to what is currently known. The strategy for continuous and more extensive PC focusing on the hotspots is required during the remaining SDG era.

## 3. Diagnosis of UGS

The recommended principal diagnosis for IS is fecal microscopy by the Kato–Katz (KK) smear, according to the WHO guidelines [1]. The KK smear can detect IS by both qualitative and quantitative screening of the fecal smear, but this method is limited by its low diagnostic sensitivity. Fortunately, a rapid test that detects a circulating cathodic antigen (CCA) or circulating anodic antigen (CAA) of *S. mansoni* can supplement the low diagnostic sensitivity of the KK fecal microscopy for IS [84,85]. Because the CCA/CAA test can detect trace amounts of an antigenic protein in urine, a positive test means active infection, the same as egg positive. Therefore, the CCA/CAA test is a useful screening of IS with a sensitivity of 89% and specificity of 55% and is especially sensitive for low-intensity infection groups of IS [84]. While CCA/CAA is known to be specific to the genus *Schistosoma*, its diagnostic sensitivity for *S. haematobium* infection is not high, with a sensitivity of 39% and specificity of 78% [84]. The CCA rapid kit is useful for field surveys of IS but not of UGS.

For the diagnosis of UGS, the global standard is UM for identifying eggs of *S. haematobium* in the urine. The microscopic detection of eggs from urine provides the only direct evidence of parasite infection and has the highest specificity. However, it is widely acknowledged that the diagnostic sensitivity of UM is low; therefore, the egg-positive rates represent the minimum confirmed rates. The actual infected population is likely higher than what is detected due to the diagnostic limitations of UM. UM is particularly less sensitive for the diagnosis of light infections caused by a low burden of infected worms, similar to the KK smear for IS, because of the irregular and uneven passage of eggs. Other indirect methods can supplement the diagnosis, such as the rapid stick test of microhematuria, serology, and ultrasound scanning for the diagnosis of UGS.

### 3.1. Urine Microscopy

The microscopic observation of urine allows for the relatively easy identification of *S. haematobium* eggs. These eggs possess distinctive size and shape characteristics that enable microscopists to identify them with relative ease, provided that the urine samples have been properly prepared. However, the diagnostic sensitivity of UM is limited due to the irregular passage of eggs into the urine.

The worms may continuously lay eggs in their habitat. Some of these eggs are carried along with the blood and flow systematically, while others become trapped near the worms, forming blood clots adjacent to the infected venule. The infected venule undergoes inflammation and swelling due to the stimulation by worms and eggs, which weakens its wall particularly during acute UGS. When the urinary bladder is full, the pressure from the bladder lumen compresses the venule wall, helping to keep it intact. However, during voiding, when the bladder is being emptied, the pressure is reversed, exerting force from the wall towards the lumen. This pressure change can cause rupturing of the inflamed, weakened, and engorged venule wall. The rupture of the venule wall during voiding leads to the release of eggs into the urine which are then discharged into the environment. Once in the environment, the eggs can continue the life cycle of *S. haematobium* through asexual reproduction and larval growth in a snail host. After the rupture, the bladder wall and venule undergo rapid tissue repair, resulting in only a few eggs being discharged through the repaired venule wall.

UM is capable of detecting eggs of *S. haematobium* only when they are present in the urine, and it is important to note that only approximately half of the eggs are passed in this manner [84,85]. The remaining eggs are not excreted but instead are deposited in nearby tissues such as the urinary bladder wall, male or female genital organs, or other organs in the body via the bloodstream [38]. This process of egg deposition leads to inflammation, granulation, fibrosis, and calcification of the affected tissues, which are characteristics of chronic UGS.

#### 3.1.1. UM by Filtration

The microscopic screening of filtered urine and morphological identification of *S. haematobium* eggs constitute the globally recommended standard diagnosis for UGS as endorsed by the WHO. According to this method, urine samples of 10 mL are filtered through a Millipore membrane and the membrane is subsequently examined under a microscope [4]. Due to their big size, the eggs can easily be observed and identified on the membrane. This method allows for both qualitative and quantitative analysis, as the number of eggs on the filter membrane can be counted. These egg counts are positively correlated with the worm burden in infected individuals.

**Figure 1:**
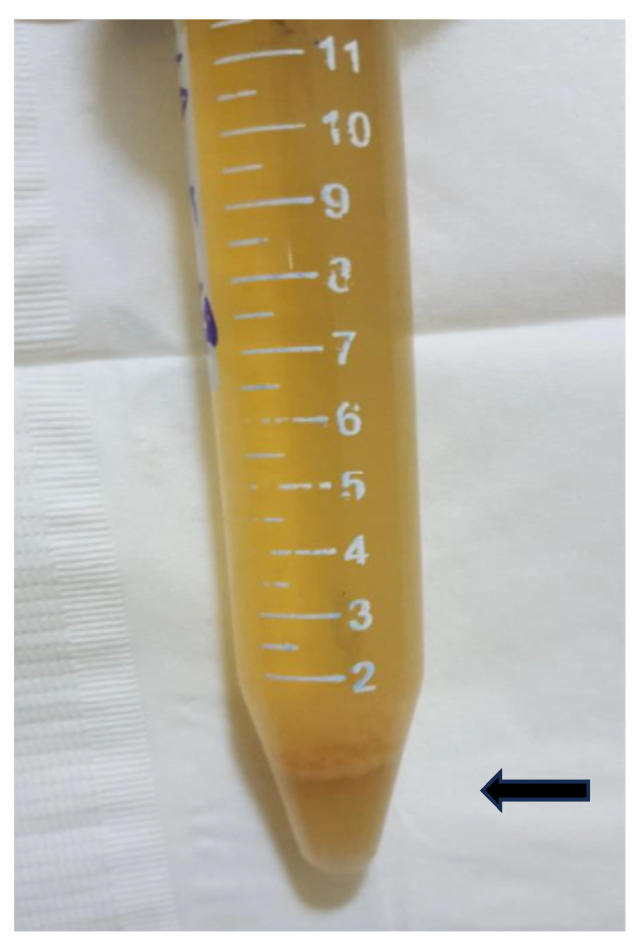
Gross view of a urine sample with sedimented sludge (arrow) of tissue debris and cells, egg-positive 14-year-old boy.

While the filtration-based UM is widely used in most surveys, it has certain limitations. The procedure necessitates the use of filter membranes and cartridges, which are produced solely by high-tech companies that may not be located in SSA. Moreover, in endemic areas, urine samples can often contain sludge materials of blood cells, blood clots, epithelial cells, and desquamated tissues, which can make the filtration process challenging (Figure 1). In such not-uncommon scenarios, routine UM by filtration becomes difficult to perform properly.

The use of repeated UM with multiple urine samples taken on consecutive days may enhance the detection of eggs, thereby addressing the low diagnostic sensitivity observed in a single UM examination. Zulu et al. (2020) [58] conducted a study in South Africa and reported that the egg-positive rate increased from 24.1% with a single urine sample examination to 34.0% when using 3-day urine samples. Another study by Midzi et al. (2020) [80] estimated the diagnostic sensitivity of a single UM to be 53.8% when employing a comprehensive gold standard that combined UM, micro- and macrohematuria, and a history of macrohematuria as positive evidence.

It is important to note, however, that this approach of multiple sampling and examination or the establishment of a comprehensive positive gold standard is feasible only in limited intensive surveys on a small scale. This is due to challenges related to compliance from both examinees and examiners. Consequently, most field surveys in practice adopt a single sampling and examination approach, as they are cross-sectional studies that adhere to the limitations of low sensitivity.

Researchers should take into account the low sensitivity of UM in prevalence surveys despite UM being the standard diagnosis of UGS. The sensitivity is influenced by the prevalence of disease and the infection burden. As the prevalence decreases, the sensitivity of diagnosing UGS also decreases. In other words, the challenge lies in the diagnosis of low or ultra-low-intensity infections of UGS, where the sensitivity of UM becomes a significant concern.

#### 3.1.2. UM by Centrifugation

In field settings, UM by centrifugation is used as an alternative to UM by filtration, primarily due to the limitations of the filtration method. Since the eggs of *S. haematobium* are relatively large and heavy, they can be easily concentrated through a short centrifugation process of approximately 5 min or by allowing the urine to stand for a specific period of time. By counting the eggs in the entire pellet obtained after centrifugation of 10 mL of urine, quantitative UM can be performed. This method has the potential to replace UM by filtration for both qualitative and quantitative examinations.

However, it is important to note that UM by centrifugation is limited by the requirement of centrifuge machines and additional time needed for the microscopy of specimens with large pellet samples. Similar to UM by filtration, it also faces the limitation of low sensitivity.

#### 3.1.3. UM by or Mobile Phone Microscopy or SchistoScope

A mobile phone microscope, recently named the “SchistoScope,” was developed for point-of-care screening and counting of *S. haematobium* eggs in urine within limited lab settings in SSA [85,86,87]. This method offers several advantages, such as using mobile phones instead of voluminous microscopes and the availability of semi-automatic programmed screening. Numerous studies have reported the development of improved devices that are more suitable for mobile lab use in the microscopic diagnosis of malaria and helminthiases.

One meta-analysis reviewed two reports on *S. haematobium* diagnosis using the SchistoScope [85]. The first report showed a diagnostic sensitivity of 35.6% (95% CI 25.9–46.4%) and a specificity of 100% (96.6–100%). The second report demonstrated a sensitivity of 72.1% (95% CI 56.1–84.2%) and a specificity of 100% (95% CI 75.9–100%) [85]. While the diagnostic specificity was comparable to that of UM, the sensitivity was lower. However, this method requires a special device to attach to the mobile phone and a light source for reading the filtration membrane [86,87].

Despite microscopic examination of specific eggs being the standard diagnosis of UGS, it is limited by low sensitivity. Particularly, light or ultra-light infections often yield false-negative results. Therefore, when interpreting data and formulating intervention strategies for elimination, it is crucial to consider these limitations.

### 3.2. Detection of Hematuria

Hematuria refers to the presence of blood in the urine, either microscopical or gross. Various diseases can cause hematuria, including malignancy, inflammations, infections, and stones in the kidney, ureter, or urinary bladder. Among these diseases, UGS is the most common cause of hematuria, particularly in SSA, both in its microscopic and gross (or macroscopic) forms.

Gross hematuria is more frequently observed in cases of acute UGS, while the urinary bleeding tends to become microscopical as the UGS progresses chronically. Consequently, the detection of hematuria has been considered as evidence of UGS in SSA, especially among children, as they rarely present with malignancies or other conditions that cause urinary bleeding. The detection of hematuria is relatively simple and easy to implement in the field setting for diagnosis, and several studies have reported its use as a diagnostic tool for UGS (Table 2 and Table 3).

#### 3.2.1. Detection of Microhematuria by Urine Reagent Strips

Microhematuria (MicH) is defined as the observation of five or more red blood cells per field using UM on a direct smear of urine, even when there are no visible signs of blood in the urine. Direct smear UM is not practical in the field; therefore, urine reagent strips, also known as urine dipsticks, are commonly used for screening purposes to detect MicH. Numerous reports have compared the rates of MicH positivity with egg positivity using urine reagent strips. Table 2 presents survey reports with UM and MicH or macrohematuria (MacH) results, while Table 3 provides reports of the diagnostic evaluation of MicH.

Overall, urine reagent strips have shown higher positive rates than UM when using the same urine specimens (Table 2 and Table 3). Among SAC, the MicH rate ranged from 13.6% with a 5.0% UM positivity [6], while it was 65.7% MicH among 1283 SAC with a 61.2% UM positivity in Angola [7]. In a mapping study of SAC in Ethiopia, the positive MicH rate was 2.8% compared to a 0.13% UM positivity [21], and among 12,102 SAC and adults, MicH was detected in 2.4% of individuals with a UM positivity of 0.2% [22]. While some studies reported lower rates of MicH compared to UM in the same surveyed subjects, these studies reported higher positive rates of MicH ranging from 0.5% to 10% (Table 2). The difference between MicH and UM positivity was more pronounced in cases of ultra-light infections. For instance, one cross-sectional survey reported a rate of 83% positive MicH cases among 400 SAC, with a UM positivity of 49.2% in Nigeria [52]. In UGS cases with heavy infections, the two methods showed little difference in their positive rates.

The survey findings indicate that a certain portion of UGS cases with hematuria are falsely negative by UM due to its low sensitivity. In SSA, it is reasonable to speculate that most of the people with hematuria may have UGS because other causes of bleeding are less common. When the UM positivity was set as the gold standard, the sensitivity of MicH by urine reagent strips ranged from 60% to 100%, with a specificity of 75.7–99.8% (Table 3). Ochodo et al. [84] reviewed 74 previous studies and estimated a sensitivity of 75% (95% confidence interval 71% to 79%) and a specificity 87% (95% confidence interval 84% to 90%). Knopp et al. [72] reported an MicH positivity of 2.7% among 37,077 egg-negative children and 71.6% among 2130 egg-positive children in Tanzania. The difference between UM and MicH positivity is more significant in ultra-light infections with 1–5 eggs/10 mL than those with other egg counts. The MicH-positive rates were 50.1% for 1–5 eggs/10 mL, 70.1% for 6–10 eggs/10 mL, 81.6% for 11–49 eggs/10 mL, and 93.0% for >50 eggs/10 mL. Among egg-positive children, 28.4% were MicH negative and 7% of children with heavy egg counts >50 tested negative [72]. These findings undoubtedly demonstrate the higher diagnostic sensitivity of one urine reagent strip test compared to UM, although there are some false-negative cases. Hematuria is not consistently present in UGS like egg passing in urine. Another study demonstrated the infection-intensity-dependent accuracy of the urine reagent strip test for the diagnosis of UGS, with 100% sensitivity observed for over 15 egg counts/10 mL [88]. Yet another study described a good sensitivity of 81% (95% CI 79–83%) and a specificity of 89% (95% CI 87–92%) of the MicH test [89]. Consequently, the detection of MicH using urine reagent strips is a choice of primary screening of UGS, and it may replace UM for the diagnosis of UGS in the field setting of SSA.

#### 3.2.2. Macrohematuria

Macrohematuria (MacH) is a condition characterized by visibly bloody urine that is recognized with the naked eye. Prevalence surveys in SSA reported MacH positivity rates from 0.3% to 3.0% in areas where the UM yielded 6.9–34.2% positive rates (Table 2). Generally, the MacH rates were reported to be 1/10 or less compared to the positive rates of UM or MicH. In a hyperendemic area, however, Bocanegra et al. (2015) [7] reported a high MacH positivity rate of 17.1% among SAC in Angola, where the egg-positive rate was 61.2% by UM and 65.7% by MicH. The diagnostic sensitivity of MacH was estimated at 27.1%, while the specificity was 97.5% in the study [7]. The highest MacH positivity was recorded at 55% at tip-villages in Chad, where the UM of subjected SAC identified a 100% positivity rate in a newly recovered hyperendemic area of UGS [13]. Despite the extraordinarily high prevalence, the area had not been noticed until the survey. Until 2015, UGS in that area had been completely neglected, and the MacH survey marked the global record of 55% positivity. In Nigeria, a MacH-positive rate of 29.7% was observed among 279 SAC, while their UM-positive rate was 67.4% [45]. These reports demonstrated extraordinarily high rates of MacH in hyperendemic areas of UGS where the UM-positive rate exceeded 60%.

Gross urinary bleeding occurs as a result of severe desquamation of the urinary bladder wall and bleeding by rupture of multiple inflamed veins in acute UGS. The acute inflammatory lesion of the bladder wall slowly progresses to a chronic state due to repeated rupture and healing of the mucosa and veins, leading to progressive fibrosis. In chronic UGS, even in cases with many eggs, the majority of urine samples appear grossly clear, indicating a low diagnostic sensitivity of MacH. In reality, most individuals with MacH have mixed lesions of acute and chronic UGS as they experience repeated super-infections in hyperendemic areas [90,91]. The high positive rate of MacH in SSA suggests a state of hyperendemicity of UGS in the survey regions.

### 3.3. Serodiagnosis and Molecular Diagnosis of UGS

#### 3.3.1. Serology

Serology is one of common diagnostic methods of infections by identifying specific antigens or antibodies. The detection of a CAA or CCA of *S. mansoni* in serum or urine by a rapid dip test is promising for the diagnosis of IS but not for UGS [92].

Mangano et al. (2020) [93] examined 288 residents in Burkina Faso by enzyme-linked immunosorbent assay (ELISA) to detect anti-SEA (secretory egg antigen) IgG antibodies in their serum and the study reported a 63% positivity rate for *S. haematobium* infection. The percentage of positive individuals increased in the age group of 5–9 years over 50% and reached a peak of nearly 90% in individuals 10–14 years and 15–19 years; it decreased to 60% in adults. The serum antibodies remained detectable longer in aged individuals, which represented cumulative exposure to the antigen rather than the current prevalence. The serology data suggested that approximately 10% of the population remained unexposed to *S. haematobium* infection in the surveyed endemic area of Burkina Faso [93]. Therefore, serologic screening of serum antibodies provides endemicity information of UGS within a certain defined area.

Song et al. (2018) [94] developed an in-house ELISA system for the diagnosis of UGS using SEA of *S. haematobium* and detecting specific serum IgG antibodies. They screened 149 Sudanese subjects and found that 27 (18.1%) were negative by both UM and ELISA, 58 (38.9%) were UM positive, 119 (79.9%) were ELISA positive, 55 (36.9%) were positive for either, 3 were UM-only positive, and 64 were ELISA-only positive. Considering UM as the gold standard, the diagnostic sensitivity of ELISA was 94.8% while the specificity was 29.7%. These findings demonstrated that ELISA for specific IgG antibodies is not a suitable choice for UGS diagnosis. The report also recorded that 18.1% of subjects were negative by both UM and ELISA, suggesting that a proportion of the subjects had not been exposed to antigenic challenge by the parasite. A review by Hinz et al. (2016) [95] summarized serological approaches for the diagnosis of all species of *Schistosoma* infection. Based on the review, ELISA for serum antibodies to SEA of *S. haematobium* showed 87–96% sensitivity and 31–32% specificity.

Serodiagnosis has limited diagnostic value with low specificity in high endemicity areas due to numerous false-positive cases after cure. However, serology data may provide valuable epidemiological information in target areas. Additionally, serodiagnosis holds potential for diagnosing UGS in non-endemic or ultra-low-endemicity settings.

Sheele et al. (2013) [96] developed a rapid diagnostic test for *S. haematobium* based on the detection of the surface IgG coat on filtered eggs with 97% sensitivity and 78% specificity. However, no further studies were reported on this method.

#### 3.3.2. Molecular Diagnosis

The detection of DNA or RNA fragments through polymerase chain reaction (PCR), quantitative PCR (qPCR), loop-mediated isothermal amplification (LAMP), and recombinase polymerase amplification (RPA) has been applied for the diagnosis of UGS. In Nigeria, PCR showed a positive rate of 34.7% in the 777 subjects while UM yielded only a 1.7% positivity rate. This significant disparity between the two methods highlights the notable difference in diagnostic outcomes [97]. Sow et al. (2023) [98] compared diagnostic values of qPCR for UGS, which produced a sensitivity of 98.9%, specificity of 81.8%, positive predictive value (PPV) of 58.1%, and negative predictive value (NPV) of 99.6%. Archer et al. (2020) [99] conducted isothermal RPA targeting the *S. haematobium* Dra1 genomic region in urine samples and assessed its diagnostic value in comparison to UM findings. The study reported a sensitivity of 93.7% (88.7–96.9%), specificity of 100% (69.1–100%), PPV of 100% (97.5–100%), and NPV of 50% (27.2–72.8%). The findings proposed RPA as a promising field-applicable diagnostic measure because it used a low-temperature isothermal reaction with minimally required equipment [99].

**Table 3:** Diagnostic evaluation of microhematuria for UGS.

Country	Urine Strip Test for Microhematuria					Gold-Standard (%)	References
	Positive Rate (%)	Sensitivity (%)	Specificity (%)	Positive Predictive Value	Negative Predictive Value		
Angola	65.7	96.0	61.3	88.8		UM 61.2	[7]
Cameroon	24.9	70.3	87.9			UM 31.5	[12]
Ethiopia	22.5	99.3	88.1	53.8	99.8	UM 12.2	[19]
Ethiopia	2.35	100	97.4			UM 0.2	[22]
Tanzania	9.3	78	99.8	97.8	97.8	UM 7.4	[62]
		0					
	48.0	96.6	82.6	77.8	97.4	UM 38.7	
Zimbabwe		81	96.9	87.2	95.2	Early SI	[78]
Zambia DDIA		60				UM 61%	[75]
IHA		74					
Meta-analysis		81	89				[89]
Tanzania	26.6	75.0	75.7	18.4	97.6	UM 6.8	[90]
Senegal	23.1					UM 20.3 qPCR 34.6	[98]

UGS = urogenital schistosomiasis, UM = urine microscopy, qPCR = quantitative polymerase chain reaction, SI = *Schistosoma* infection, DDIA = dipstick dye immunoassay, IHA = indirect hemagglutination.

Gandasegui et al. (2015) [100] developed a LAMP assay called the Rapid-Heat LAMPellet method targeting *S. haematobium* ribosomal intergenic spacer DNA sequences. The study used 94 urine samples and amplified their extracted DNA with primer pairs to show 100% sensitivity and 86.67% specificity.

Overall, several molecular diagnosis methods have been developed and all of them have shown higher sensitivity than UM and MicH. The methods can serve as a reliable gold standard for the diagnosis of UGS as they exhibit almost 100% sensitivity. It is well known that UM is not sufficiently sensitive as a gold standard of UGS diagnosis; therefore, the gold standard is a limitation of the evaluation of any diagnostic measure. However, the molecular methods are limited in the field setting due to the requirements of sophisticated reagents, complex procedures, and specialized equipment. The methods are warranted in a well-established laboratory mainly for research purposes.

### 3.4. Ultrasound Scanning

In the year 2000, the WHO convened the Second International Workshop, during which a guideline was developed to standardize the morbidity assessment of UGS using ultrasound images [101]. Field researchers have utilized sonography to supplement UGS diagnosis in endemic areas following the WHO guideline.

Kim et al. (2016) [102] analyzed sonography images of 948 subjects and proposed inclusion of the “echogenic snow sign” in the urinary bladder as an additional sonographic finding for diagnostic criteria of UGS. The study was conducted in Sudan, where UM identified a 14.0% positive rate for *S. haematobium* eggs. Sonography of the same subjects demonstrated a positivity rate of 16.4% according to the old WHO criteria, and a positivity rate of 20.9% when the new criteria, which included the echogenic snow sign, were applied [102]. The study estimated the diagnostic sensitivity of ultrasound to be 81.6% using the old criteria and 85.0% using the new criteria. When the gold standard was defined as a positive result by either UM or ultrasound, the diagnostic sensitivity of UM was 70.0% according to the old criteria and 57.1% according to the new criteria. These findings provide strong evidence supporting the diagnostic enhancement provided by sonography in overcoming the low sensitivity of UM.

**Figure 2:**
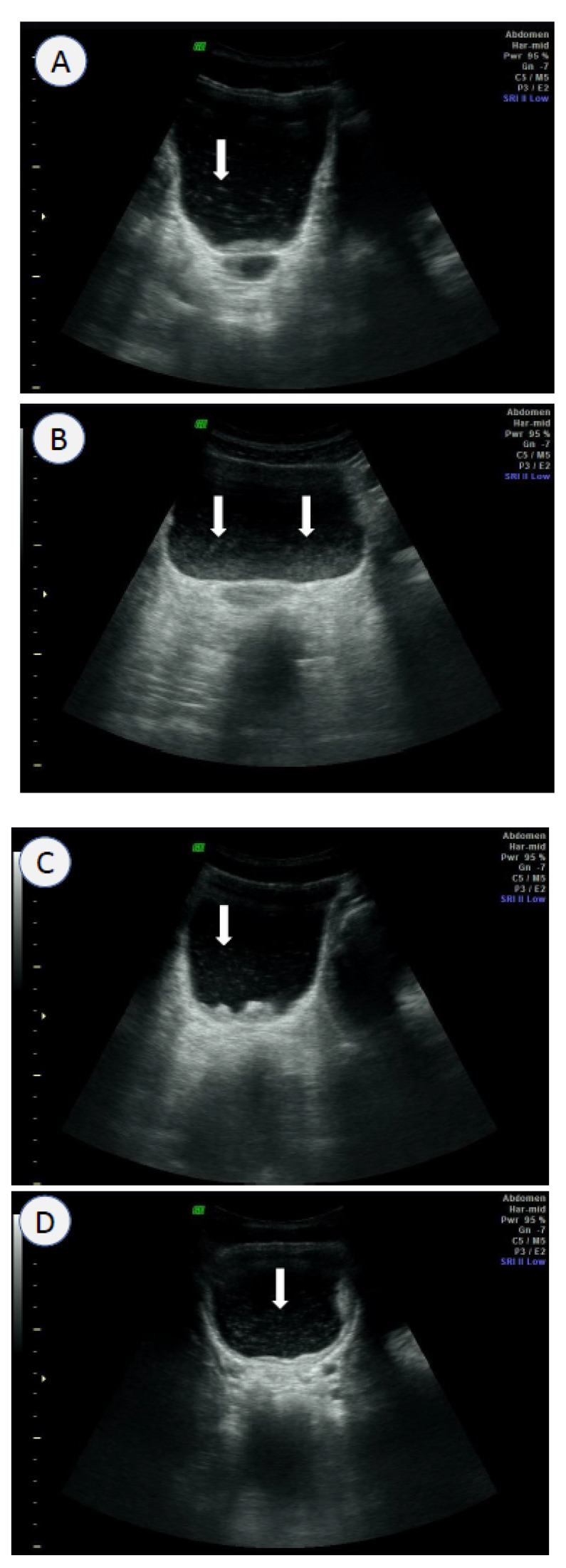

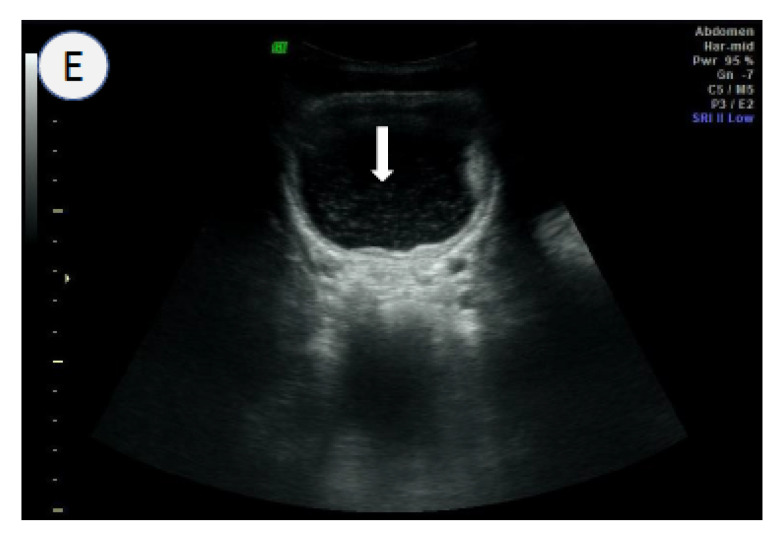
Ultrasound images of the urinary bladder in *S. haematobium* egg-positive Sudanese with the echogenic snow sign (arrows), wall thickening, and/or mass: (**A**) 14-year-old boy; (**B**) 18-year-old girl; (**C**) 20-year-old man; (**D**) 6-year-old girl; (**E**) 5-year-old-girl.

The term “echogenic snow sign” was coined to describe bright sonographic flares observed within the urinary bladder. The flares are caused by tissue debris and agglutinated blood cells suspended in the urine (Figure 2). The bladder wall in UGS undergoes pathological changes of inflammation, thickening, tumor or polyp formation, and calcification. The changes lead to desquamation of the bladder wall and/or blood vessels during expansion and contraction of the bladder. The resulting tissue debris and desquamated cells become suspended in the urine and appear as echogenic flares during sonography. The urine containing necrotic tissue and cells looks grossly turbid and/or bloody and echogenic by ultrasound (Figure 1 and Figure 2).

Sonography is influenced by the subjective interpretation of images, requiring well-trained sonographers and expensive portable equipment. However, despite these limitations, this technique provides precious information of the bladder pathologies and morbidity related to UGS. Ngome (2020) [103] recommended sonography as a useful diagnostic tool in Africa to differentiate morbidities and to ensure proper management of patients’ conditions. In a study in Angola, Bocanegra et al. (2018) [104] followed up 70 SAC by ultrasound scanning 6–8 months after praziquantel medication. Their images showed improvement in 53 SAC (75.7%), no changes in 12 (17.1%), and progression in 5 (7.1%) [105]. The use of ultrasound allows for the practical and beneficial management of infected cases of UGS by providing non-invasive and safe screening of the pathological consequences and outcomes after medication.

Santos et al. (2015) [105] compared ultrasound images and cystoscopy findings of 80 subjects with UGS in Angola. They highlighted the diagnostic significance of identifying the distorted shape of the urinary bladder [105]. Cystoscopy can directly visualize pathological changes of the urinary bladder, but it is limited by its invasive nature. Cozzi et al. (2020) [106] collected various ultrasound images of UGS as a pictorial essay, which may be reference images for diagnostic image criteria.

In this context, ultrasound scanning of the urinary bladder serves as a valuable diagnostic tool of UGS in the field setting. This method offers the advantages of being non-invasive and providing real-time information on the pathological changes associated with UGS. Furthermore, ultrasound scanning may produce other abdominal morbidities together, such as liver pathologies by IS. The diagnostic sensitivity of ultrasound is higher than that of UM, but it is important to update the old WHO criteria to include additional findings, such as the echogenic snow sign [102] and distorted contour of the urinary bladder [105]. However, it is essential to note that ultrasound diagnosis requires well-trained sonographers and the use of expensive portable ultrasound equipment, which can be a limitation in certain settings.

## 4. Conclusions

The present review summarized recent reports on UGS prevalence in SSA, mostly by using UM of SAC. There were some mapping studies that reported prevalence rates ranging from 0.13% in Ethiopia to 17.56% in Benin, but many cross-sectional surveys reported a wide range of UGS prevalence, often much higher than those reported in the mapping studies (Table 2). Furthermore, most of the reports were conducted before the COVID-19 pandemic. During the pandemic, most ongoing schistosomiasis control programs in SSA had to be halted or cancelled, which may have resulted in an actual increase in UGS prevalence compared to what is currently known. This situation is likely to cause a delay in the elimination of UGS as outlined in the original roadmap set by the World Health Organization (WHO). More aggressive surveys and intensive control activities are required to eliminate UGS in SSA by 2030.

Table 4 summarizes advantages, limitations, and a cost–benefit analysis of the currently used diagnostic methods of UGS. The diagnosis of UGS is crucial for any program, but there is no single diagnostic method that is sensitive, specific, and practical enough for the field setting. The current diagnostic methods for UGS have their advantages and limitations, as described in Table 4. The global standard for UGS diagnosis is the detection of eggs through UM, but its diagnostic sensitivity is low, especially in cases of ultra-light infections. The use of a urine reagent strip may complement or replace UM by detecting microhematuria with acceptable sensitivity and specificity. I propose the urine reagent strip as an alternative standard method for UGS diagnosis in the field setting in SSA. Ultrasound may also be included in the field program for UGS to monitor morbidity on site, but the diagnostic criteria should be updated including the echogenic snow sign and deformity. There are several options available for UGS diagnosis in the field setting, and researchers can choose the most suitable methods based on the purpose of the survey.

## Figures and Tables

**Table 1:** WHO reports on preventive chemotherapy for schistosomiasis in sub-Saharan African countries, 2021.

Countries	No. of Treated People	No. of Treated SAC	SAC Coverage (%)	National Coverage (%)
Angola	2,025,995	2,025,995	62.6	40.0
Benin	755,772	755,772	79.5	28.6
Botswana	364,502	364,502	86.3	25.7
Burkina Faso	1,154,320	870,624	89.9	89.9
Burundi	1,681,868	1,681,868	105.5	96.5
Cameroon	2,766,864	2,766,864	83.7	51.1
Central African Republic	106,940	106,940	68.4	6.5
Chad	1,887,341	1,887,341	91.3	45.5
Congo	79,859	79,859	70.2	18.5
Côte d’Ivoire	2,328,781	1,904,844	85.4	56.7
Democratic Republic of Congo	11,885,901	11,146,568	93.4	67.8
Egypt (2020)	2,897,891	1,448,102	98.9	98.8
Eritrea	374,853	168,590	95.3	82.8
Ethiopia	3,562,162	3,071,245	84.2	23.0
Gambia	63,011	37,607	46.6	13.6
Ghana	2,792,184	2,792,184	79.5	24.1
Guinea	2,192,543	2,192,543	91.8	45.4
Guinea-Bissau	83,515	83,515	99.8	50.7
Kenya	3,165,901	1,374,465	95.2	80.0
Liberia	489,715	489,715	84.8	35.3
Madagascar (2020)	2,705,026	2,676,453	80.4	27.3
Malawi	2,780,558	1,431,603	82.6	30.6
Mali	3,977,709	3,896,892	93.9	42.3
Mauritania	141,370	141,370	86.7	15.2
Mozambique	1,125,297	864,811	95.6	6.5
Namibia (2019)	143,383	143,383	70.7	57.4
Niger	6,943,175	2,748,014	92.8	65.2
Nigeria	1,810,420	1,433,132	49.0	6.6
Rwanda	2,856,299	1,027,010	98.4	94.8
Sao Tome and Principe	17,322	17,322	90.2	42.8
Senegal	1,845,926	1,482,743	95.6	64.1
Sierra Leone (2020)	1,071,836	678,929	61.1	35.7
Somalia (2020)	2,549,993	2,549,993	113.7	89.6
South Sudan	12,461	8,067	66.1	0.4
Sudan (2019)	3,058,201	2,483,677	55.0	37.9
Togo	463,782	174,469	94.6	11.3
Uganda (2020)	5,301,075	2,628,166	84.1	53.0
Tanzania	9,374,223	8,209,186	82.2	51.6
Zambia	1,925,614	1,925,614	80.0	43.8
Zimbabwe (2020)	404,006	317,617	79.0	11.8

Source: WHO. *Control of Neglected Tropical Diseases*. PCT Databank—Schistosomiasis, 2023 [4]. SAC = school-aged children.

**Table 2:** Summary of prevalence reports of UGS by UM and hematuria in sub-Saharan Africa countries after 2018.

Countries	Studies	Subjects	No. of Examined	Positive Rate, % by			Year of Survey	Year of Publish	References
				UM	MicH	MacH			
Angola	Mapping	SAC	31,938	12.6			2018-9	2022	[5]
	Cross-sectional	SAC	17,093	5.0	13.6			2022	[6]
	Cross-sectional	SAC	1283	61.2	65.7	17.1	2013-4	2015	[7]
Benin	Mapping	SAC	19,250	17.56			2013-5	2019	[8]
Burkina Faso	Cross-sectional	SAC	228	0				2021	[9]
Cameroon	Cross-sectional	Men	89	31.4				2022	[10]
	Cross-sectional	SAC	389	32.6	24.4		2018	2021	[11]
	Cross-sectional	Community residents	778	31.5	24.9		2018	2021	[12]
	Cross-sectional	Adults	509	18.7	16.5		2019	2021	[13]
Chad	Cross-sectional	SAC	11,832	100		55	2015-9	2022	[14]
	Cross-sectional	SAC + Ad	258	39.2	58.9		2019	2022	[15]
Côte d’Ivoire	Cross-sectional	SAC	170	20.6				2023	[16]
	Cohort/	SAC + Ad	12,239	13.1			2015-9	2022	[17]
	Cross-sectional	Ad	901	1.0				2022	[18]
Ethiopia	Cross-sectional	SAC	1171	12.2	22.5			2022	[19]
	Cross-sectional	SAC	1288	31.6	32.1		2021-2	2022	[20]
	Mapping	SAC	15,133	0.13	2.8		2018-9	2022	[21]
	Cross-sectional	SAC + Ad	12,102	0.2	2.4			2022	[22]
Gabon	Cross-sectional	SAC	451	26.3				2021	[23]
	Longitudinal	SAC	328	43				2019	[24]
	Longitudinal	SAC	739	30.3			2012-4	2018	[25]
Gambia	Cross-sectional	SAC	2016	10.2	18.0	0.5	2015	2021	[26]
	Mapping	SAC	10,434	4.2			2014	2021	[27]
Ghana	Cross-sectional	SAC	336	12.8				2022	[28]
	Cross-sectional	SAC	520	6.5				2022	[29]
	Cross-sectional	SAC + Ad	114	22.8				2022	[30]
	Cross-sectional	SAC	309	10.4				2021	[31]
	Cross-sectional	SAC	469	21.1				2021	[32]
	Cross-sectional	SAC + PSAC	493	1.6				2021	[33]
Kenya	Cross-sectional	Women	534	3.8			2018	2022	[34]
	Cross-sectional	SAC + Ad	897	3.2					[35]
	Cross-sectional	Health center visitors	451	15.1			2018	2020	[36]
Madagascar	Cross-sectional	SAC	1958	30.5			2015	2016	[37]
Malawi	Cross-sectional	Fisherman	129	20.9				2022	[38]
	Cross-sectional	SAC	240	24.0			2019	2020	[39]
Mozambique	Cross-sectional	SAC	19,039	60.4			2011	2018	[40]
Niger	Cross-sectional	SAC + Ad	48,192	15.7			2011	2020	[41]
			54,451	8.8			2015	2020	[41]
Nigeria	Cross-sectional	SAC	250	15.2				2023	[42]
	Cross-sectional	SAC	5514	7.1			2019	2022	[43]
	Cross-sectional	Pastoral community		34.2		2.5		2022	[44]
	Cross-sectional	SAC	279	67.4		29.7		2022	[45]
	Cross-sectional	SAC	777	1.7	11.6		2015-6	2022	[46]
	Cross-sectional	SAC + Ad	432	28.9			2020	2022	[47]
	Cross-sectional	SAC	487	34.1				2022	[48]
	Cross-sectional	SAC	1113	13.7	13.7			2021	[49]
	Cross-sectional	SAC	2023	10.4				2021	[50]
	Cross-sectional	SAC	466	19.1				2021	[51]
	Cross-sectional	SAC	400	49.2	83.0			2021	[52]
	Cross-sectional	SAC	400	17.3	22.0			2021	[53]
	Cross-sectional	SAC	251	65.3				2018	[54]
Senegal	Cross-sectional	Sac	821	66.7			2018	2021	[55]
	Cross-sectional	SAC Ad	1285 300	54.2 32.0			2016-8	2020	[56]
South Africa	Cohort	SAC	1976	16.9			2007-8	2020	[57]
	Cross-sectional	SAC	970	32.2			2010	2020	[58]
South Sudan	Cross-sectional	SAC	13,286	3.7			2016-9	2022	[59]
Sudan	Longitudinal	SAC		9.1/35.2				2020	[60]
	Mapping	SAC	100,726	5.2			2016-7	2019	[61]
Tanzania	Cross-sectional	SAC + PSAC	20,389	7.4	9.3	0.3	2019	2022	[62]
	Hospital	Women	216	2.3			2021	2022	[63]
	Cross-sectional	SAC	396	5.8				2022	[64]
	Cross-sectional	SAC	649	52.7	46.2	13.1	2021	2022	[65]
	Cross	PSAC	385	16.9	17.9	6	2021	2022	[66]
	Cross-sectional	Adolescents	433	15.9		3.0		2022	[67]
	Cross-sectional	SAC	1288	31.6	32.1		2021-2	2022	[68]
	Cross-sectional	SAC	389	6.9	9.5	1.3		2022	[69]
	Cross-sectional	SAC+ PSAC	1560	0.83	0.9			2021	[70]
	Cross-sectional	Women RA	426	4.5			2019	2020	[71]
	Mapping	Population-based subjects	17,280	5.3			2006-7	2020	[72]
	Cross-sectional	SAC Adult	39,207 18,155	5.4 2.7	6.5		2013-6	2018	[73]
Zambia	Cross-sectional	SAC	173	3.5				2022	[74]
	Cross-sectional	SAC	421	9.7				2022	[75]
	Cross-sectional	SAC	243,148	61				2020	[76]
Zimbabwe	Cross-sectional	PSAC	136	22.1				2022	[77]
	Cross-sectional	Pregnant women	4437	10.6	24.4			2021	[78]
	Longitudinal	PSAC	204	19.6			2019	2021	[79]
	Cross-sectional	SAC	361	26.8				2020	[80]
	Cross-sectional	SAC	12,656	17.6			2010-1	2020	[81]
	Cross-sectional	PSAC	416	35.1				2020	[82]

UGS = urogenital schistosomiasis, UM = urine microscopy, MicH = microhematuria, MacH = macrohematuria, SAC = school-aged children, Ad = adults, PSAC = school-aged children.

**Table 4:** Advantages, limitations, and cost–benefit analysis of current diagnostic measures of UGS in the field setting.

Diagnosis	Advantages	Limitations	Cost–Benefit Analysis
Urine microscopy	Standard method Familiar in most endemic areas High specificity (96.6–100%)	Low sensitivity (25.9–46.4%) Very low sensitivity in ultra-light infections (<10%)	Good Cheap cost Feasible
CCA/CAA	High sensitivity (89%) and moderate specificity (55%) for intestinal schistosomiasis Good for field surveys in areas with ultra-light infection	Reliable for *S. mansoni* infections Not acceptable for diagnosis of UGS Cross reactions with other types of schistosomiasis Limited supply of the kit	Moderate Moderate cost Most feasible in the field setting for IS
Urine reagent strips for microhematuria	High sensitivity (71–79%) High specificity (84–90%) Easy to implement	Not standard, supplementary to UM	Good Cheap cost Most feasible in the field setting for UGS
Gross screening of macrohematuria	Cheap Easy High specificity Reflects acute infections	Low sensitivity (<10%) with numerous false-negative cases	Most cheap
Serology (ELISA)	High sensitivity (87–96%) Optimal in non-endemic areas	Low specificity (31–32%) Not practical in the endemic field setting in SSA	Moderate Moderate cost Lab facilities required
Molecular diagnosis (PCR, qPCR, and LAMP)	High sensitivity (>90%) High specificity (>95%) Optimal for setting gold standard Expensive reagents and high technique	Well-established lab support, not practical in the field setting Feasible for small-scale surveys	Poor Expensive method and feasible only in lab
Ultrasound	High sensitivity (>80%) High specificity (>80%) Morbidity information of UGS on site Detection of other co-morbidities	Experienced sonographer Expensive sonograph Feasible for small-scale surveys	Poor Expensive method Feasible with a portable sonograph

UGS = urogenital schistosomiasis, CCA = circulating cathodic antigen, CAA = circulating anodic antigen, UM = urine microscopy, ELISA = enzyme-linked immunosorbent assay, PCR = polymerase chain reaction, qPCR = quantitative polymerase chain reaction, LAMP = loop-mediated isothermal amplification.

## Abbreviations

CAA	circulating anodic antigen
CCA	circulating cathodic antigen
ELISA	enzyme-linked immunosorbent assay
IS	intestinal schistosomiasis
KK	Kato–Katz
LAMP	loop-mediated isothermal amplification
MacH	macrohematuria
M&E	monitoring and evaluation
MDA	mass drug administration
MDGs	millennium development goals
MicH	microhematuria
NGO	non-governmental organization
NTDs	neglected tropical diseases
PC	preventive chemotherapy
PCR	polymerase chain reaction
qPCR	quantitative polymerase chain reaction
RPA	recombinase polymerase amplification
SAC	school-aged children
SCI	schistosomiasis control initiative
SDGs	sustainable developmental goals
SEA	soluble egg antigen
SSA	sub-Saharan Africa
UGS	urogenital schistosomiasis
UM	urine microscopy
UN	United Nations
WHO	World Health Organization
